# Population structure and identification of genomic regions associated with productive traits in five Italian beef cattle breeds

**DOI:** 10.1038/s41598-024-59269-z

**Published:** 2024-04-12

**Authors:** Daniele Colombi, Giacomo Rovelli, Maria Gracia Luigi-Sierra, Simone Ceccobelli, Dailu Guan, Francesco Perini, Fiorella Sbarra, Andrea Quaglia, Francesca Maria Sarti, Marina Pasquini, Marcel Amills, Emiliano Lasagna

**Affiliations:** 1 Department of Agricultural, Food and Environmental Sciences (DSA3), University of Perugia, Borgo XX Giugno 74, 06121 Perugia, Italy; 2 Centre for Research in Agricultural Genomics (CRAG), CSIC-IRTA-UAB-UB, Campus Universitat Autonòma de Barcelona, Carrer de la Vall Moronta, 08193 Bellaterra de Cerdanyola del Vallés, Spain; 3 Department of Agricultural, Food and Environmental Sciences (D3A), Università Politecnica delle Marche, 60131 Ancona, Italy; 4 Department of Animal Science, University of California, Davis, CA 2251 USA; 5 Department of Agronomy, Food, Natural Resources, Animals and Environment, University of Padova, 35020 Legnaro, Italy; 6 National Association of Italian Beef-Cattle Breeders (ANABIC), 06132 San Martino in Colle, Perugia Italy; 7 Department of Animal and Food Science, Universitat Autònoma de Barcelona, 08193 Bellaterra, Spain

**Keywords:** Animal breeding, Genetic association study, Genetic markers, Genomics

## Abstract

Italy has a long history in beef production, with local breeds such as Marchigiana, Chianina, Romagnola, Maremmana, and Podolica which produce high-quality meat. Selection has improved meat production, precocity, growth ability and muscle development, but the genetic determinism of such traits is mostly unknown. Using 33K SNPs-data from young bulls (N = 4064) belonging to these five Italian breeds, we demonstrated that the Maremmana and Podolica rustic breeds are closely related, while the specialised Marchigiana, Chianina, and Romagnola breeds are more differentiated. A genome-wide association study for growth and muscle development traits (average daily gain during the performance test, weight at 1 year old, muscularity) was conducted in the five Italian breeds. Results indicated a region on chromosome 2, containing the myostatin gene (*MSTN*), which displayed significant genome-wide associations with muscularity in Marchigiana cattle, a breed in which the muscle hypertrophy phenotype is segregating. Moreover, a significant SNP on chromosome 14 was associated, in the Chianina breed, to muscularity. The identification of diverse genomic regions associated with conformation traits might increase our knowledge about the genomic basis of such traits in Italian beef cattle and, eventually, such information could be used to implement marker-assisted selection of young bulls tested in the performance test.

## Introduction

Italy has a long tradition in beef cattle production and local breeds such as Marchigiana (MAR), Chianina (CHI), Romagnola (ROM), Maremmana (MRM), and Podolica (POD) produce a high-quality lean meat with low level of subcutaneous and intermuscular fat, as a result of three major contributing factors: genetics, feeding, and farming management. These breeds are light-coated although new-born calves are wheat-coated, and they are distributed in Central to Southern Italy. Genetic selection in Italian beef cattle is implemented by the National Association of Italian Beef Cattle Breeders (ANABIC) and aims to improve meat production, precocity, growth ability, and muscle development^1^. Three of the five Italian beef cattle breeds under the ANABIC breeding management, MAR, CHI, and ROM, are highly specialised in beef production, while the other two, MRM and POD, are considered rustic breeds^2–4^. The specialised breeds are reared both on semi-extensive or intensive systems, while the rustic ones are selected for adaptability to harsh environments. MAR, CHI and ROM are bred to produce labelled meat, with the protected geographical indication (PGI, “Vitellone Bianco dell’Appennino Centrale”), which is exclusively produced along the Apennine mountains of Central Italy^5^, according to the specification approved by EU^6^. The description and the geographical distribution of the five breeds under investigation are reported in Supplementary File S1 and Supplementary Fig. S1, respectively. Current selection programs, based on the traditional quantitative approach, have achieved a remarkable improvement of growth, daily weight, and muscularity gain. Moreover, cattle are somatically well-developed with a correct morphology and light skeletal apparatus^7,8^. In addition, the selection scheme of MRM and POD enhances the maintenance of traits, such as conformation, growth, and coat colour, that are important for their environmental adaptation. Morphometric, growth, and muscularity traits have moderate to large heritabilities^9^, indicating the existence of an important genetic component.

Genome-wide association studies (GWAS) based on large numbers of single nucleotide polymorphisms (SNPs) have made possible to identify genomic regions associated with growth and muscularity phenotypes in beef cattle^10–12^, leading to the detection of a high number of quantitative trait loci (QTL) that are gathered in the Cattle QTL database^13^. Several GWAS for growth and muscularity traits have been performed in beef cattle. An et al.^12^ detected candidate genes associated with body measurements in Chinese Wagyu beef cattle. They found several SNPs within or near 11 candidate genes underlying the phenotypic expression of hip height, body height, and body length. Similarly, 37 significant SNPs and several important candidate genes were associated with body weight in Chinese Simmental beef cattle^14^. Moreover, GWAS has been successfully applied to detect QTLs and candidate genes for complex phenotypes in beef cattle, such as morphometric traits in Beninese indigenous cattle breeds^10^, carcass traits in Chinese Simmental beef cattle^15^, and liveweight traits in Braunvieh cattle breed^16^. Up to date, only few GWAS for beef production traits have been carried out in Italian cattle breeds. Sorbolini and colleagues^17^ detected 96 markers significantly associated with carcass and meat traits in 409 Marchigiana bullocks, using an Illumina 50K BeadChip assay. Besides, Pegolo and colleagues^18^ performed a GWAS analysis in a sample of 1166 double-muscled Piemontese beef cattle identifying 37 significant SNPs associated with 12 carcass and meat quality traits.

The main goals of the current study were to characterize the diversity and population structure of the five Italian beef cattle breeds (MAR, CHI, ROM, MRM, and POD) and to identify genomic regions associated with the phenotypic variation of growth and muscularity traits recorded in these populations.

## Results and discussion

### Characterization of growth and muscularity phenotypes recorded in the five Italian breeds

Two phenotypic traits, average daily gain (ADG) during performance test, and weight at 1 year old (WEI), were evaluated in each breed, while muscularity (MUS) was measured only in the three specialised breeds (MAR, CHI, and ROM). Means and standard deviations for recorded phenotypic traits are shown in Table 1. The highest ADG value was observed in the CHI breed. Consequently, also the highest WEI was recorded in the CHI breed. MRM and POD bulls had lower average ADG and WEI than those observed in MAR, CHI, and ROM. As expected, MUS was higher in MAR (411.8, linear score), because of the double muscling phenotype segregating in this breed (i.e., 656 normal, 235 hypertrophic, and 20 unknown genotypes in the final 911 MAR dataset used for further analysis).

**Table 1:** Mean and standard deviation (in parentheses) of phenotypes recorded in five Italian beef cattle breeds.

Phenotype	Marchigiana (N = 911)	Chianina (N = 937)	Romagnola (N = 916)	Maremmana (N = 366)	Podolica (N = 571)
ADG (kg/d)	1.621 (0.242)	1.734 (0.237)	1.591 (0.230)	1.483 (0.258)	1.303 (0.201)
WEI (kg)	543.6 (51.5)	583.2 (50.8)	524.3 (54.9)	412.6 (58.7)	365.6 (57.7)
MUS (score)	411.8 (63.3)	386.9 (57.6)	384.6 (58.6)	–	–

*N* number of observations, *ADG (kg/day)* average daily gain during performance test, *WEI (kg)* weight at 1 year of age, *MUS (score)* muscularity.

### Genetic diversity and population structure of the five Italian breeds

**Figure 1:**
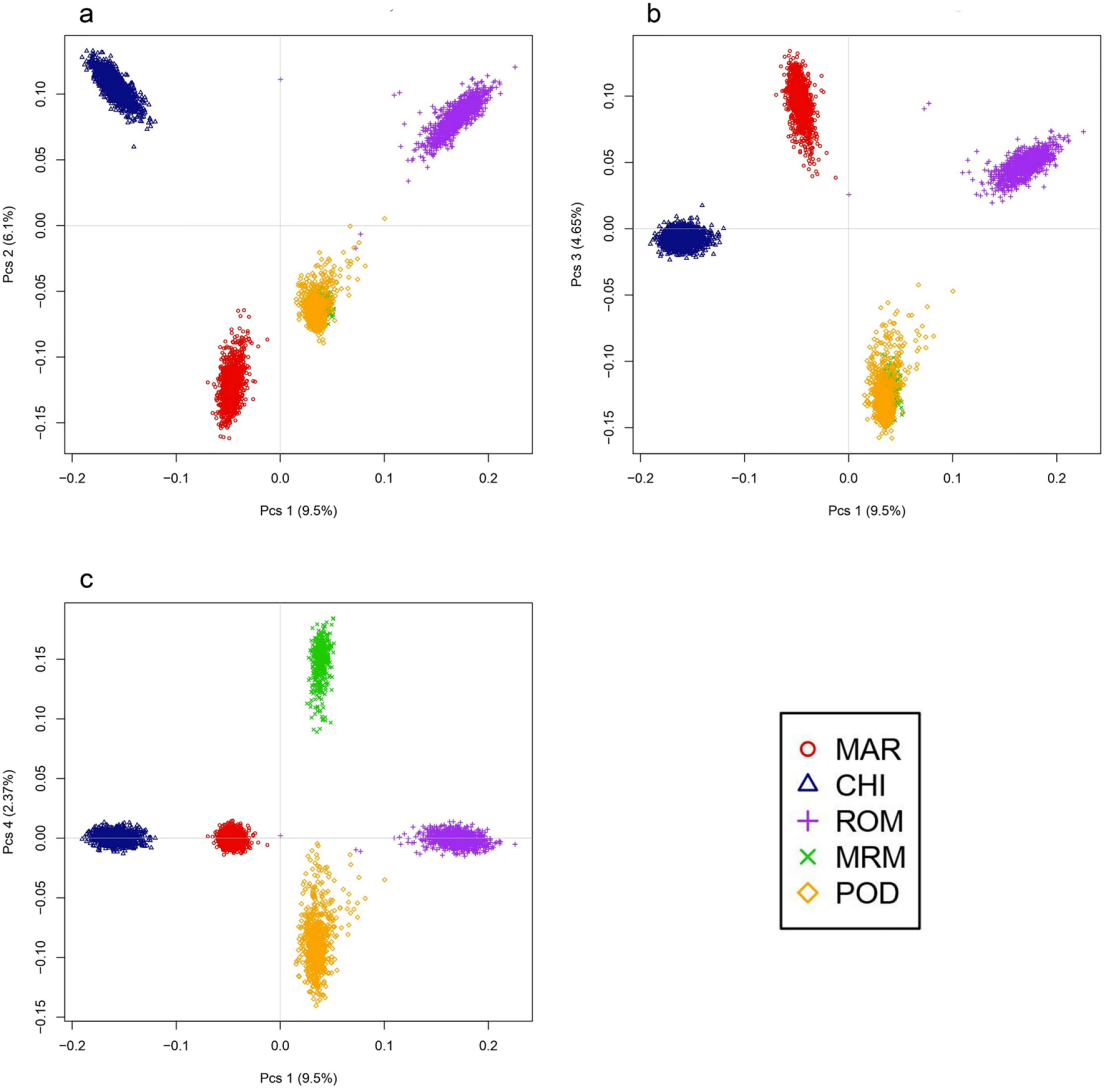
Principal Component Analysis plots of the five Italian breeds. (**a**) PC1 vs PC2, (**b**) PC1 vs PC3, (**c**) PC1 vs PC4.

In the principal component analysis (PCA), the first component explained 9.5% of the genetic variance and separated CHI from ROM, while MAR lies precisely between these two breeds, an observation that agrees well with the ethnological origin of MAR (Supplementary Description S1) In contrast, the second component explained 6.1% of the genetic variance and separated MAR from CHI and ROM. The rustic breeds (MRM and POD) are closely related, since they group together at the centre of the graph (Fig. 1a). PC3 (4.65%) discriminated MAR from the rustic breeds (Fig. 1b), which become fully separated by PC4 (2.37%) (Fig. 1c).

**Figure 2:**
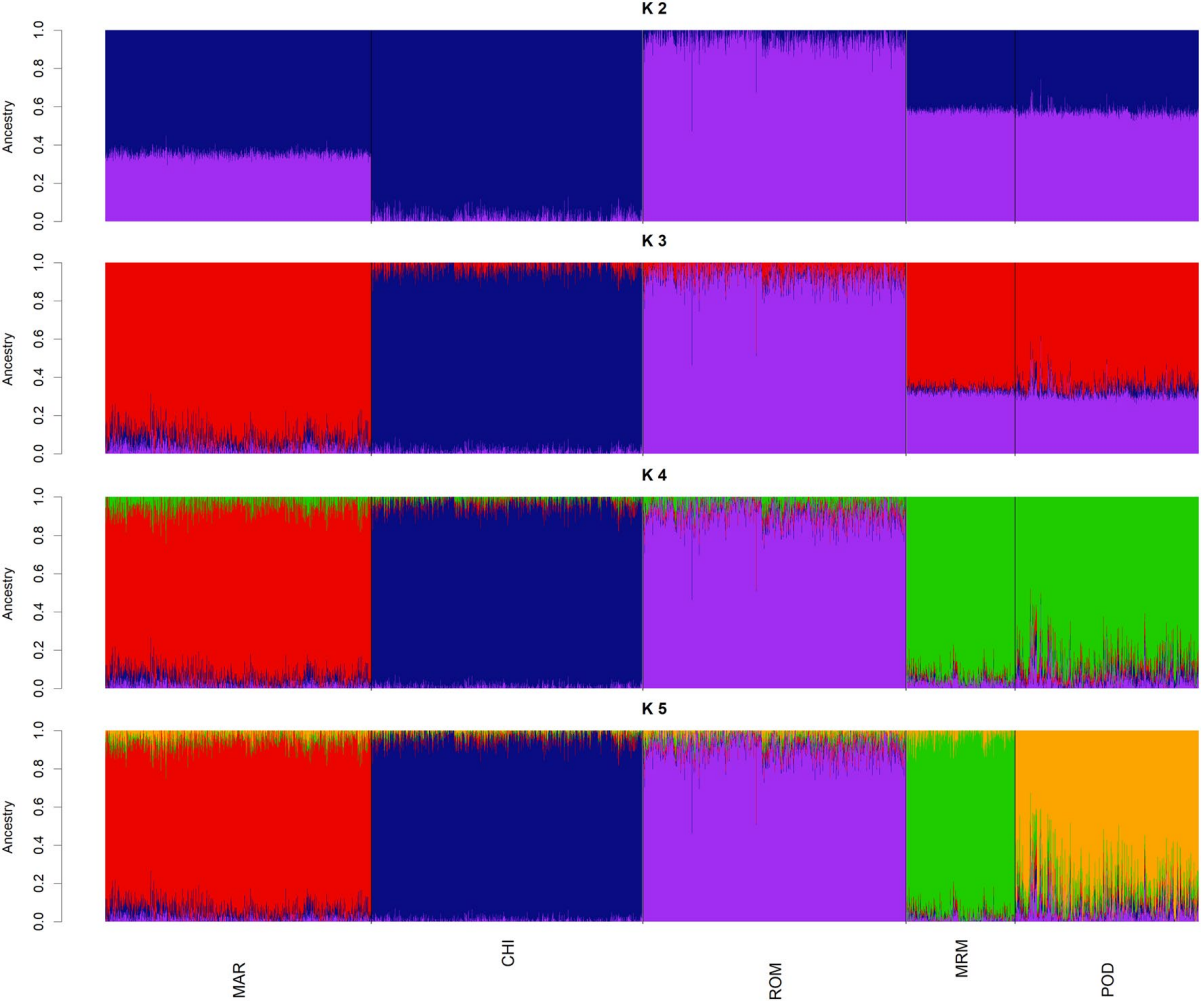
Admixture plots of the five Italian breeds with *K* from 2 to 5. Population structure comprised the following clusters: MAR (*K* = 3), CHI and ROM (*K* = 2), MRM (*K* = 4), POD (*K* = 5).

The pairwise *F*_ST_ coefficients amongst breeds were generally low, indicating that they are weakly differentiated (Table 2). The highest pairwise *F*_ST_ value (0.077) was obtained between ROM and CHI, while the lowest value (0.053) corresponded to MRM vs POD, thus confirming the high genetic similarity between these two rustic breeds (Table 2). Admixture analyses were consistent with the PCA and *F*_ST_ analyses by showing that POD and MRM are the two most closely related breeds. At *K* = 2 a cluster between ROM and CHI was observed, MAR clustering mimicked the distribution shown in PC1, possibly confirming its CHI and ROM crossbreeding origins. MAR showed its genetic distinctiveness at *K* = 3, a number of clusters at which each specialised breed (MAR, CHI, and ROM) was genetically differentiated from the rustic ones, which still grouped together. Indeed, POD and MRM only become clearly differentiated at *K* = 5, which is the *K*-value with the lowest cross-validation (CV) error (Fig. 2).

**Table 2:** Pairwise *F*_ST_ estimates between the five Italian cattle breeds.

Breed	Marchigiana	Chianina	Romagnola	Maremmana	Podolica
Marchigiana	–				
Chianina	0.063	–			
Romagnola	0.066	0.077	–		
Maremmana	0.064	0.076	0.068	–	
Podolica	0.057	0.067	0.063	0.053	–

Genetic differences between specialised and rustic breeds were expected because they differ in terms of breeding history, farming systems, and breeding programs^19^. Selection plans for the rustic breeds are characterized by a low selection intensity due to the need of having sufficient males for natural service. Moreover, their geographical distribution is quite limited, i.e., Tuscany and Lazio (Central Italy) for MRM, and Basilicata, Calabria and Puglia regions (Southern Italy) for POD (Supplementary Fig. S1). Furthermore, POD and MRM are raised in extensive farming system, in small or medium-sized herds and generally they are fed on pasture all year long. In contrast, specialised breeds (MAR, CHI, and ROM) are scattered throughout Central and Southern Italy regions, and they have been intensively selected throughout their history, being mainly housed in intensive conditions^20^. In the study carried out by Mastrangelo and colleagues^21^, considering 32 Italian cattle breeds, the MAR, CHI, ROM, MRM, and POD breeds were ascribed to the Podolian trunk, appearing as closely related in the PCA analysis. The low pairwise *F*_ST_ coefficients measured in the present study (Table 2) are consistent with the ones reported in the literature^21^, pointing out to an extensive sharing of alleles probably due to the recent ancestry of breeds deriving from the Podolian trunk.

A close relationship between POD and MRM was already observed by Mastrangelo and colleagues^21^. According to Moioli and colleagues^22^, the POD and MRM breeds have a common ancestor belonging to the Grey Steppe group of cattle characterised by a grey coat color and long horns. However, the origin of the Podolian cattle is still a matter of debate, with many alternative hypotheses suggesting that they might come from Podolia (western Ukraine) or they might have dispersed from the eastern steppe in direction to Anatolia, the Balkans, and Italy^19,23^. It is even possible that Podolian breeds are derived from Near Eastern bovine populations that arrived 3–5 kya BP to Central Italy through the Mediterranean Sea corridor^24^. Although MAR, CHI, ROM, MRM, and POD share a common ancestry, the proportions of the different genetic backgrounds that contributed to their formation as well as the different selection pressures might explain the weak genetic differentiation observed in the present investigation.

### Genome-wide association study for productive traits

**Figure 3:**
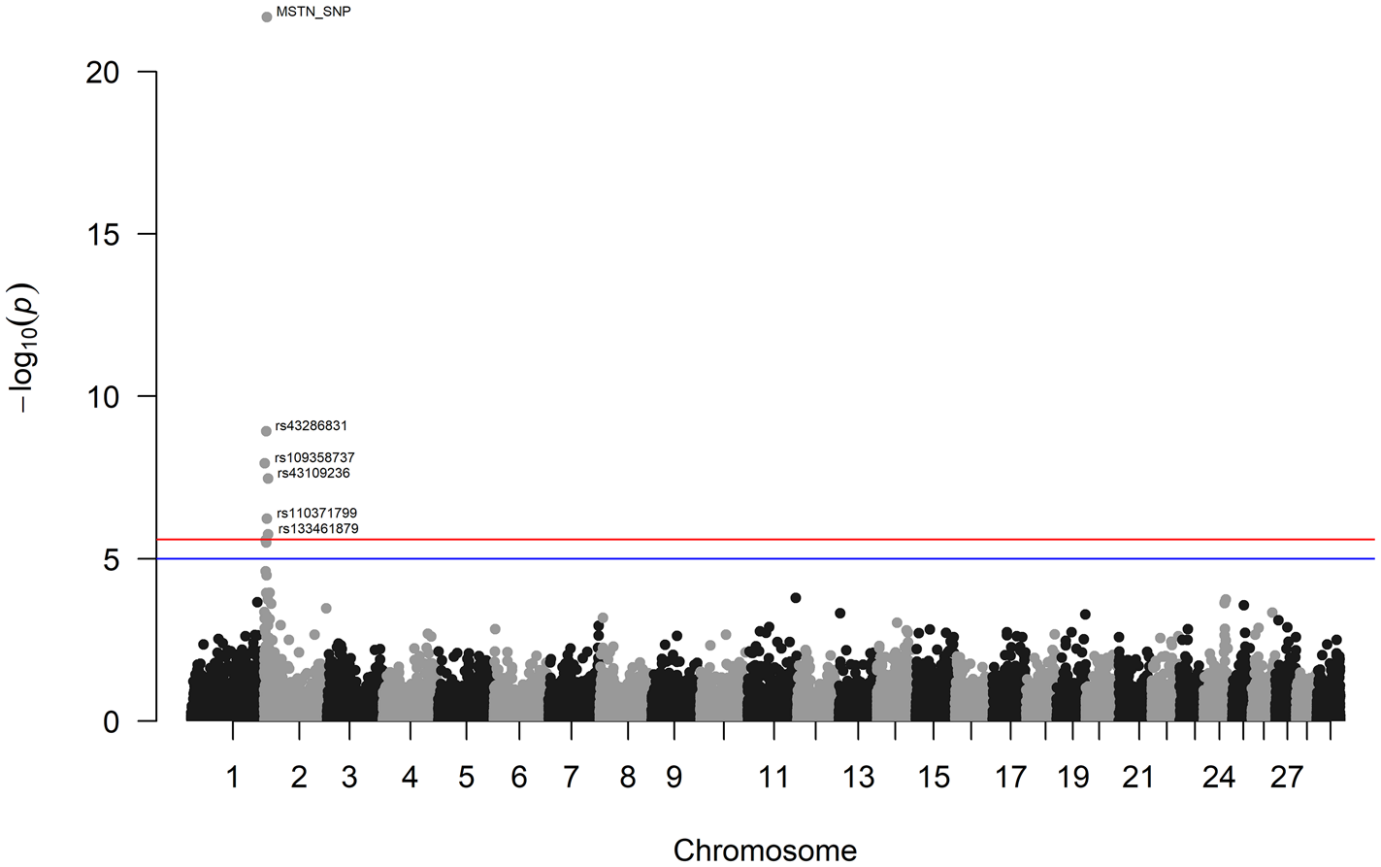
Genome wide significant associations between SNPs and muscularity in Marchigiana breed. Negative log_10_ *P*-values (Y-axis) of the association between SNPs and the muscularity are plotted against the genomic location of each SNP marker (X-axis). The red line represents the Bonferroni-corrected threshold of significance, while the blue line represents the suggestive threshold of significance (*P*-value of 0.05).

Genome-wide significant associations were detected in the current work between MUS phenotype and polymorphisms segregating in the MAR and CHI breeds. Regarding the MAR breed, six SNPs exceeded the threshold of significance level on chromosome (BTA) 2 (Table 3, Fig. 3, Supplementary Fig. S2a).

**Table 3:** Markers associated with muscularity in the Marchigiana and Chianina breeds.

Genome-wide significant associations								
rs	BTA	bp	A1	MAF	β	SE (*β*)	*P*-value	Candidate genes^a^
Marchigiana								
MSTN_SNP	2	6283727	T	0.132	0.9037	0.0887	3.64E−23	*MSTN*
rs43286831	2	4636218	A	0.296	0.3858	0.0628	1.22E−09	*AMMECR1L, SFT2D3, LIMS2, MYO7B, SAP130, UGGT1, HS6ST1*
rs109358737	2	1283089	G	0.235	0.4146	0.0720	1.14E−08	*TUBGCP5, IMP4, PTPN18, AMER3, ARHGEF4, CYFIP1, NIPA1, NIPA2, HERC2*
rs43109236	2	8826383	A	0.227	0.3979	0.0714	3.37E−08	*TFPI, CALCRL*
rs110371799	2	5909758	G	0.450	0.2904	0.0577	5.73E−07	*MFSD6, NAB1, INPP1, NEMP2, HIBCH, C2H2orf88, MSTN*
rs133461879	2	8634840	A	0.280	0.3262	0.0678	1.75E−06	*TFPI, CALCRL*
Chianina								
rs41624840	14	27376642	A	0.467	0.2557	0.0869	1.27E−06	*ASPH, CLVS1, NKAIN3*

*rs* SNP identifier according to the Ensembl database, *BTA* *Bos taurus* autosome, *bp* position in base pairs, *A1* minor allele, *MAF* minor allele frequency, *β* allelic substitution effect, *SE (β)* standard error of β.

^a^Genes previously associated to growth and productive traits in cattle in a range of 0.5 Mbp from the significant SNP.

This large chromosomal region on BTA2 (1.2–8.8 Mb) contains the myostatin (*MSTN*) locus and other neighbouring genes that have important roles in muscle differentiation and development, as reported by Doyle and colleagues^25^. In 1997, the discovery of the causal mutation explaining the double-muscled phenotype in several bovine breeds, such as Belgian Blue, Asturiana de los Valles and Maine-Anjou, was a crucial step towards understanding the key role of the *MSTN* gene in the development of muscle hypertrophy^26^. The muscular hypertrophy phenotype segregates in the MAR breed due to a mutation at nucleotide 871 in exon 3 (ENSBTAT00000015674.6:c.871G>T, represented by Ensembl sequence ENSBTAT00000015674.6) in the *MSTN* gene^27^. This point mutation has a remarkable effect on the myostatin protein, changing a codon for glutamic acid into a stop codon (E291X variant) that blocks the translation of 257 bases of the third exon. The variant MSTN_SNP (*P*-value 3.640819e−23) is indeed such causative mutation and confirms the implication of the third exon in the proper functioning of myostatin because it encodes the C-terminal region that is fundamental for the protein tridimensional folding^27^. Myostatin is a negative regulator of muscle growth, so its inactivation leads to muscle hypertrophy (double muscling)^28,29^.

The double muscling phenotype can be beneficial from an economic point of view for the increased muscle mass, dressing percentage, meat tenderness, and a reduction in meat collagen content^30^. In this regard, Ceccobelli and colleagues^5^ reported higher values of hot carcass weight and dressing yield in heterozygous bulls than in the ones homozygous for the wild allele. However, extreme muscle hypertrophy is undesirable because it is associated with macroglossia, hypoplasia of vital organs, dystocia, etc. Therefore, the genetic management of hypertrophy can differ among breeds and countries^31^. Among the main autochthonous beef cattle breeds reared in Italy, the double-muscling phenotype is only segregating in MAR and Piemontese cattle^2,18^. In MAR the frequency of the *MSTN* mutation is low, probably due to the exclusion of homozygous animals from mating plans^3,32^.

Other candidate genes were identified through the analysis of the gene content of genomic regions showing associations with muscularity. For instance, the rs43286831 marker (BTA2: 4.63 Mb) mapped in a range of 0.5 Mbp from the AMMECR1 like (*AMMECR1L*), SFT2 domain containing three (*SFT2D3*), LIM zinc finger domain containing two (*LIMS2*), myosin VIIB (*MYO7B*), Sin3A associated protein 130 (*SAP130*), UDP-glucose glycoprotein glucosyltransferase 1 (*UGGT1*), and heparan sulfate 6-O-sulfotransferase 1 (*HS6ST1*) genes. Variation near or within the *AMMECR1L*, *MYO7B, SAP130, UGGT1,* and *HS6ST1* loci has been associated to carcass traits, conformations, weight and fatness phenotypes^33^. The *SFT2D3* and *LIMS2* genes have been also associated to fatness in the Hanwoo breed^34^. Besides, polymorphism in the *LIMS2* gene was associated with carcass traits^35^. Finally, *MYO7B* has been proposed as candidate gene involved in the development of the hind quarter^25^. The same region harbours also different genes (i.e. *WDR33*, *GPR17*, *IWS1*, *PROC*) which were not previously associated with growth or muscle development.

In the BTA2: 1.28 Mb region, the rs109358737 marker mapped near to the tubulin gamma complex component 5 (*TUBGCP5*), IMP U3 small nucleolar ribonucleoprotein 4 (*IMP4*), protein tyrosine phosphatase non-receptor type 18 (*PTPN18*), APC membrane recruitment protein 3 (*AMER3*), Rho guanine nucleotide exchange factor 4 (*ARHGEF4*), cytoplasmic FMR1 interacting protein 1 (*CYFIP1*), NIPA magnesium transporter 1 (*NIPA1*), NIPA magnesium transporter 2 (*NIPA2*), and HECT and RLD domain containing E3 ubiquitin protein ligase 2 (*HERC2*) genes. Involvement of *TUBGCP5*, *IMP4, PTPN18,* and *AMER3*^35^ and *ARHGEF4*, *NIPA1* and *NIPA2*^33^ in the variation of carcass traits has been reported^33,35^, while the *CYFIP1* gene has implicated in growth and meat traits in cattle^36^ and carcass weight in Charolais breed^37^. *NIPA1* and *NIPA2* are candidate genes for the development of the inner thigh in Limousin cattle^25^. Moreover, *HERC2* gene was associated to growth and meat production^36^.

The rs43109236 marker (BTA2: 8.82 Mb) is located within the tissue factor pathway inhibitor (*TFPI*) gene and rs133461879 (BTA2: 8.63 Mb) maps close to *TFPI* and calcitonin receptor like receptor (*CALCRL*) genes. In humans, *TFPI* is involved in coagulation inhibition and proliferation of vascular smooth muscle cells^38^.

Finally, the rs110371799 marker (BTA2: 5.90 Mb) mapped in the proximity of the major facilitator superfamily domain containing six (*MFSD6*), NFGI-A binding protein 1 (*NAB1*), inositol polyphosphate-1-phosphatase (*INPP1*), nuclear envelope integral membrane protein 2 (*NEMP2*), 3-hydroxyisobutyryl-CoA hydrolase (*HIBCH*), chromosome 2 C2orf88 homolog (*C2H2orf88*), and *MSTN* genes. The *MFSD6* and *NAB1* genes were previously associated to muscularity in cattle^25,39^ and *INPP1* was reported to influence swine meat quality^40^. In contrast, *NEMP2*, *HBICH*, and *C2H2orf88* were instead associated to muscularity and growth traits of different avian species^41–43^. *BIN1* gene also mapped in this region, but no previously associations were reported.

**Figure 4:**
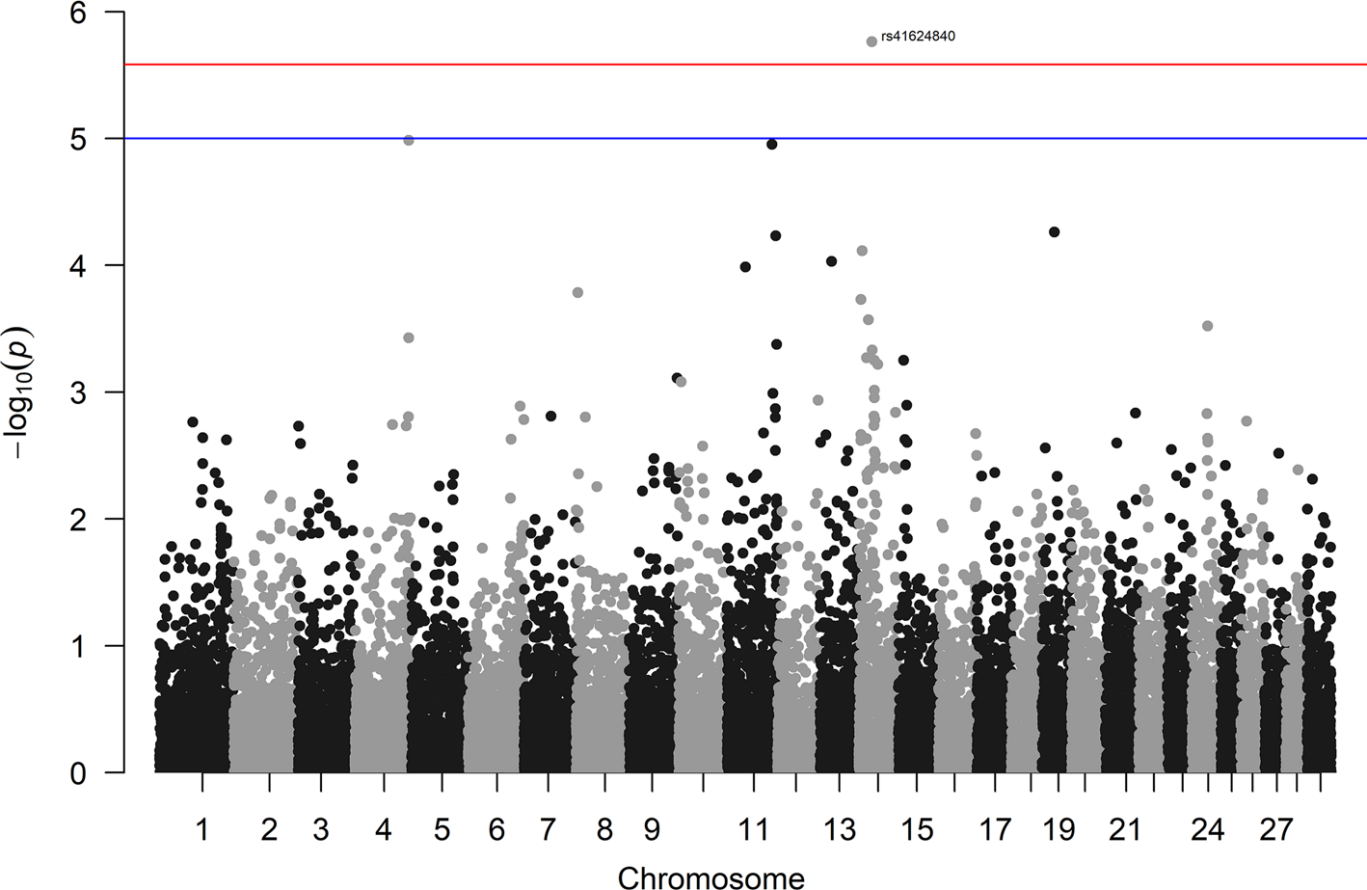
Genome wide significant associations between SNPs and muscularity in Chianina cattle. Negative log_10_ *P*-values (Y-axis) of the association between SNPs and muscularity are plotted against the genomic location of each SNP marker (X-axis). The red line represents the Bonferroni-corrected threshold of significance, while the blue line represents the suggestive threshold of significance (*P*-value of 0.05).

In the CHI breed, a SNP has been significantly associated to muscularity on BTA14 (Table 3, Fig. 4, Supplementary Fig. S2b). This SNP is located close (less than 0.5 Mbp) to aspartate beta-hydroxylase (*ASPH*), clavesin 1 (*CLVS1*)*,* and sodium/potassium transporting ATPase interacting three (*NKAIN3*) genes. The *ASPH* gene has been associated with muscular development^44^ and muscle hypertrophy^45^, being also involved in birth weight in Nelore cattle^11^ as well as in growth and development in Chinese Simmental beef cattle^46^ and Hereford and Braford breeds^47^. The *CLVS1* gene has been implicated in muscle development in Red Angus breed^48^ and in carcass and meat traits in two sheep breeds ^49,50^; while a role of the *NKAIN3* locus in growth traits has been reported in Hanwoo cattle^51,52^, as well as in sheep^49^.

No significant associations were observed between SNP genotypes and muscularity in the Romagnola breed. Similarly, no association was observed either with ADG and WEI traits.

Our results highlighted a high genetic similarity among the five Italian beef cattle breeds, and especially between Maremmana and Podolica breeds, probably due to recent ancestry. Moreover, the genome-wide association analyses revealed several genes associated to muscularity in the MAR and CHI breeds, thus demonstrating that variation in the *MSTN* gene has a very strong effect on muscularity in Marchigiana breed. Such information could be used in marker assisted selection schemes to improve meat and carcass traits in the breeds under investigation.

## Methods

### Samples collection and ethical approval

The collection of blood samples was made as established in the FAO guidelines for the characterization of animal genetic resources. Animal management and phenotype recording were made in accordance with the criteria defined in the Welfare Quality Project (WQP)^53^. All activities were approved in 2020 by the ANABIC Central Technical Committee of the National Herd-book. This approval took into consideration all aspects involved in blood collection, management, and handling of the animals. Blood sampling tasks were carried out by trained veterinarians, who adhered to standard procedures and relevant national guidelines to ensure appropriate animal care. The research was carried out in adherence to the guidelines and regulations outlined in the ARRIVE guidelines (https://arriveguidelines.org).

### Phenotypic data

The study included 4064 young bulls representing five Italian beef cattle breeds: MAR (N = 991), CHI (N = 1007), ROM (N = 979), MRM (N = 406), and POD (N = 681). Blood samples were collected by ANABIC at the genetic station of San Martino in Colle (Perugia, Italy) during the performance test from 1985 to 2022. Individual blood samples were collected from the jugular veins of the young bulls at the end of the performance test period. Samples were collected in EDTA K_3_ coated vacuum tubes and stored at − 20 ℃ prior to use. The 4064 animals represent the whole set of elite bulls available for the five breeds selected by ANABIC (until the end of year 2022).

### Phenotypic recording

All animals used in the current study were bulls in performance test. Bulls were pre-selected by evaluating morphological traits with the “new visual assessment scoring system”, which considers the adequacy to the breed standard, muscularity, dimensions, and general morphology^54^. Individuals reaching a minimum score of 75 are enrolled in the Herd-book of the breed^55^. Young bulls to be evaluated in the ANABIC genetic station must comply with three conditions: (i) their father must be an approved bull; (ii) their mother must be qualified as sire’s mother, with a score equal or higher than 82 with the “new visual assessment scoring system”; (iii) young bulls must have a pedigree verified by DNA parentage testing^55^.

Phenotypic traits of the five investigated breeds were collected at the ANABIC genetic stations during the performance test. Three phenotypic traits were evaluated:

- Average daily gain, ADG (kg/day), evaluated in all the five studied breeds;
- Weight at one year old, WEI (kg), evaluated in all the five studied breeds. This trait reflects the weight of the bulls at the end of the performance test, which lasts for 6 months;
- Muscularity, MUS (score), recorded by using a visual assessment scoring system from 1 to 5 levels, with the only exception for MAR cattle (range 1–6 in case of muscular hypertrophy). This trait was evaluated only in MAR, CHI, and ROM breeds by three trained assessors. The MUS trait is recorded by evaluating the main muscular regions of the animal (withers, shoulders, back, loins, rump, legs, and buttock) and indicates the aptitude to produce muscular tissue. The mean value recorded for each region is weighted by a specific weighting coefficient (related to the economic incidence of each commercial cut), being subsequently multiplied by 100 and included in the final muscularity genetic index (ranging from 0 to 600).

Means and standard deviations were calculated on the final number of animals used in the GWAS analyses. The normality of phenotypic data was checked with the Shapiro–Wilk test^56^. Non-normal data (i.e. data for the three specialized breeds) were rank-based transformed^57^ with the GenABEL package^58^ in R v4.0.5 for GWAS analysis.

### Genomic DNA extraction and high-throughput genotyping

Genomic DNA was extracted using the GenElute Blood Genomic DNA kit (Sigma Aldrich, St. Louis, MO, USA) as previously described by Sarti and colleagues^2^. All 4064 bulls were genotyped with the GeneSeek Genomic Profiler Bovine LDv4 33K chip (Illumina Inc., San Diego, CA, USA), which contains 30,111 SNPs, at the Agrotis Laboratory (LGS, Cremona, Italy) using standard multi-sample protocols and reagents according to the manufacturer’s instructions. This chip is the official array used by ANABIC to genotype all the young bulls evaluated in performance test. The map positions of SNPs were inferred from the ARS-UCD_1.2 bovine genome assembly^59^. By using the software PLINK v1.9^60^, SNP names and positions were updated. Prior to statistical analysis, SNP data were filtered, using the BITE package^61^ in R v4.0.5, according to the following criteria: (i) SNPs with call rates less than 95%, (ii) minor allele frequencies less than 5%, (iii) missing genotypes more than 5%, and iv) SNPs with highly significant deviation from the Hardy–Weinberg equilibrium (*P*-value < 10^−6^)^62^ were eliminated. After quality control, 980 MAR (19,762 SNPs), 1000 CHI (19,111 SNPs), 970 ROM (19,402 SNPs), 399 MRM (20,063 SNPs), and 677 POD (20,584 SNPs) remained for further analysis. Genotyped animals that did not have phenotypic recordings were removed. Thus, the final numbers of animals used for GWAS were 911 MAR, 937 CHI, 916 ROM, 366 MRM, and 571 POD.

### Population structure analysis

Principal Component Analysis was performed with the BITE package^61^ in R v4.0.5; Pairwise *F*_ST_ coefficients^63^, performed on each single autosomal variant with the method proposed by Weir and Cockerham^64^, were computed using the HIERFSTAT package^65^ in R v4.0.5, on a representative subset of 300 animals per breed, obtained via the *representative.sample()* function on the BITE package^61^ in R v4.0.5, which maintain the total original genomic variability and structure. The ADMIXTURE v1.3.0 software^66^ was used to calculate maximum likelihood estimates of individual ancestries from SNP data. The optimal *K*-value was the one with the lowest cross-validation error, as determined with the method described by Alexander and Lange^67^. The ADMIXTURE results were visualised using BITE package^61^ in R v4.0.5.

### Genome-wide association study

The GEMMA software v0.98.5^68^ was used to perform the GWAS for the five recorded traits in the five breeds under investigation. A univariate linear mixed model was fit for each trait as follows:

$$y=W\alpha +x\beta +u+\varepsilon $$

where $y$ is an n-vector of beef phenotypes for 911 MAR, 937 CHI, 916 ROM, 366 MRM, and 571 POD; $W=({w}_{1},\dots ,{w}_{c})$ is a $n\times c$ matrix of two fixed effects (plus a column of intercept with values of 1 s) including birth year (26 levels for MAR, 29 levels for CHI, 24 levels for ROM, 20 levels for MRM, and 16 levels for POD) and month of birth (12 levels for MAR, CHI, ROM, and 8 levels for MRM and POD); $\alpha$ is a c-vector of the corresponding coefficients including the intercept; $x$ is an *n*-vector of marker genotypes; $\beta$ is the effect size of the marker; $u$ is an *n*-vector of random individual genetic effects with a normal distribution $u\sim N\left(0, \lambda {\tau }^{-1}K\right)$, where ${\tau }^{-1}$ is the variance of the residual error, $\lambda$ is the ratio between the two variance components, and $K$ is the relatedness matrix derived from SNP genotypes; $\varepsilon$ is an n-vector of errors, being $\varepsilon \sim MV{N}_{n}(0,{\tau }^{-1}{I}_{n})$, where ${I}_{n}$ is an $n\times n$ identity matrix and $MV{N}_{n}$ denotes the n-dimensional multivariate normal distribution. Population structure was corrected by considering a relatedness matrix. The method of Bonferroni^69^ was implemented in order to adjust for multiple testing. The R software v4.0.5 was used to perform Manhattan plots depicting the results of the GWAS and quantile–quantile plots using qqman package^70^. Lambda genomic inflation factors (λ) were calculated with the median method (1 df) implemented in GenABEL^58^.

## Supplementary Information

Supplementary Information.

## Data Availability

All the data supporting the results of this article are displayed in the article or in the Supplementary Information. The raw phenotypic and genotypic data are stored in the drive cloud of Department of Agricultural, Food and Environmental Sciences (DSA3)—University of Perugia and can be provided by the corresponding author on reasonable request.
