# Author Correction: Population structure and identification of genomic regions associated with productive traits in five Italian beef cattle breeds

**DOI:** 10.1038/s41598-025-97766-x

**Published:** 2025-04-16

**Authors:** Daniele Colombi, Giacomo Rovelli, Maria Gracia Luigi-Sierra, Simone Ceccobelli, Dailu Guan, Francesco Perini, Fiorella Sbarra, Andrea Quaglia, Francesca Maria Sarti, Marina Pasquini, Marcel Amills, Emiliano Lasagna

**Affiliations:** 1 Department of Agricultural, Food and Environmental Sciences (DSA3), University of Perugia, Borgo XX Giugno 74, 06121 Perugia, Italy; 2 Centre for Research in Agricultural Genomics (CRAG), CSIC-IRTA-UAB-UB, Campus Universitat Autonòma de Barcelona, Carrer de la Vall Moronta, 08193 Bellaterra de Cerdanyola del Vallés, Spain; 3 Department of Agricultural, Food and Environmental Sciences (D3A), Università Politecnica delle Marche, 60131 Ancona, Italy; 4 Department of Animal Science, University of California, Davis, CA 2251 USA; 5 Department of Agronomy, Food, Natural Resources, Animals and Environment, University of Padova, 35020 Legnaro, Italy; 6 National Association of Italian Beef-Cattle Breeders (ANABIC), 06132 San Martino in Colle, Perugia Italy; 7 Department of Animal and Food Science, Universitat Autònoma de Barcelona, 08193 Bellaterra, Spain

Correction to: *Scientific Reports* 10.1038/s41598-024-59269-z, published online 12 April 2024

The original version of this Article contained errors. In the Results and discussion section, under the subheading ‘Genome-wide association study for productive traits’,

“The muscular hypertrophy phenotype segregates in the MAR breed due to a mutation at nucleotide 874 in exon 3 (g.874G > T) in the *MSTN* gene^27^. This point mutation has a remarkable effect on the myostatin protein changing, a codon for glutamic acid into a stop codon (E291X variant), that blocks the translation of 254 bases of the third exon. The variant rs3423130174 (*P*-value 3.640819e−23) is indeed such causative mutation and confirms the implication of the third exon in the proper functioning of myostatin because it encodes the C-terminal region that is fundamental for the protein tridimensional folding^27^.”

now reads:

“The muscular hypertrophy phenotype segregates in the MAR breed due to a mutation at nucleotide 871 in exon 3 (ENSBTAT00000015674.6:c.871G>T, represented by Ensembl sequence ENSBTAT00000015674.6) in the *MSTN* gene^27^. This point mutation has a remarkable effect on the myostatin protein, changing a codon for glutamic acid into a stop codon (E291X variant) that blocks the translation of 257 bases of the third exon. The variant MSTN_SNP (*P*-value 3.640819e−23) is indeed such causative mutation and confirms the implication of the third exon in the proper functioning of myostatin because it encodes the C-terminal region that is fundamental for the protein tridimensional folding^27^.”

Additionally, in Table 3, the position for Marchigiana breed in the top row was incorrect,

“rs3423130174 / 2 / 6283726 / T / 0.132 / 0.9037 / 0.0887 / 3.64E−23 / *MSTN*”

now reads:

“MSTN_SNP / 2 / 6283727 / T / 0.132 / 0.9037 / 0.0887 / 3.64E−23 / *MSTN*”.

As a result of this error, Figure 3 was incorrect. The original Figure 3 and accompanying legend appear below.

This correction does not affect the results and conclusions of this article.

The original Article has been corrected.

**Fig. 3:**
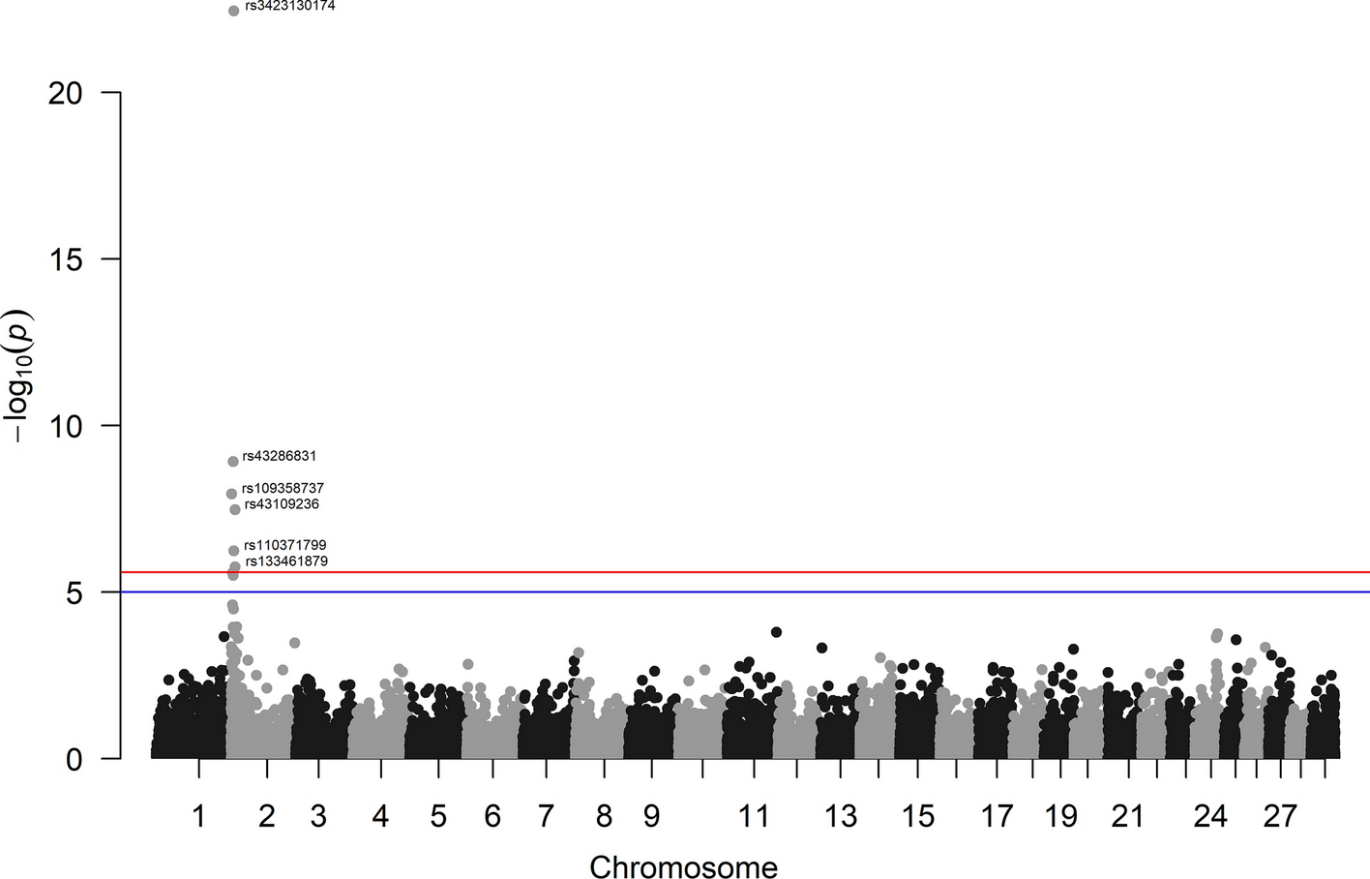
Genome wide significant associations between SNPs and muscularity in Marchigiana breed. Negative log_10_ *P*-values (Y-axis) of the association between SNPs and the muscularity are plotted against the genomic location of each SNP marker (X-axis). The red line represents the Bonferroni-corrected threshold of significance, while the blue line represents the suggestive threshold of significance (*P*-value of 0.05).

