# Supplementary material for: Population structure and identification of genomic regions associated with productive traits in five Italian beef cattle breeds
Source: Sci Rep. 2024 Apr 12;14:8529. doi: 10.1038/s41598-024-59269-z (PMC11014930; doi:10.1038/s41598-024-59269-z)
Supplement: Supplementary file 1 — Supplementary Information. [file 41598_2024_59269_MOESM1_ESM.pdf]

SUPPLEMENTARY INFORMATION FOR

Population structure and identification of genomic regions associated with productive traits in five Italian beef cattle breeds

Daniele Colombi[1], Giacomo Rovelli[1,2], Maria Gracia Luigi-Sierra[2], Simone Ceccobelli[3], Dailu Guan[2,4], Francesco Perini[5], Fiorella Sbarra[6], Andrea Quaglia[6], Francesca Maria Sarti[1], Marina Pasquini[3], Marcel Amills[2,7*+], and Emiliano Lasagna[1*+]

[1] Department of Agricultural, Food and Environmental Sciences (DSA3), University of Perugia, 06121 Perugia, Italy

[2] Centre for Research in Agricultural Genomics (CRAG), CSIC-IRTA-UAB-UB, Campus Universitat Autonòma de Barcelona, 08193 Bellaterra, Spain

[3] Department of Agricultural, Food and Environmental Sciences (D3A), Università Politecnica delle Marche, 60131 Ancona, Italy

[4] Department of Animal Science, University of California, 2251 Davis, California, United States of America

[5] Department of Agronomy, Food, Natural resources, Animals and Environment, University of Padova, 35020 Legnaro, Italy

[6] National Association of Italian Beef-Cattle Breeders (ANABIC), 06132 San Martino in Colle, Perugia, Italy

[7] Department of Animal and Food Science, Universitat Autònoma de Barcelona, 08193 Bellaterra, Spain

*Corresponding authors

Prof. Emiliano Lasagna; emiliano.lasagna@unipg.it; Department of Agricultural, Food and Environmental Sciences (DSA3), University of Perugia, Borgo XX Giugno 74 – 06121 - Perugia, Italy.

Prof. Marcel Amills; marcel.amills@uab.cat; Centre for Research in Agricultural Genomics (CRAG), CSIC-IRTA-UAB-UB, Campus Universitat Autonòma de Barcelona, Carrer de la Vall Moronta – 08193 – Bellaterra de Cerdanyola del Vallés, Spain.

[+]These authors contributed equally to the work.

Supplementary Figure S1. Geographical distribution of the five beef cattle breeds (Marchigiana, Chianina, Romagnola, Maremmana, and Podolica) in Italy.

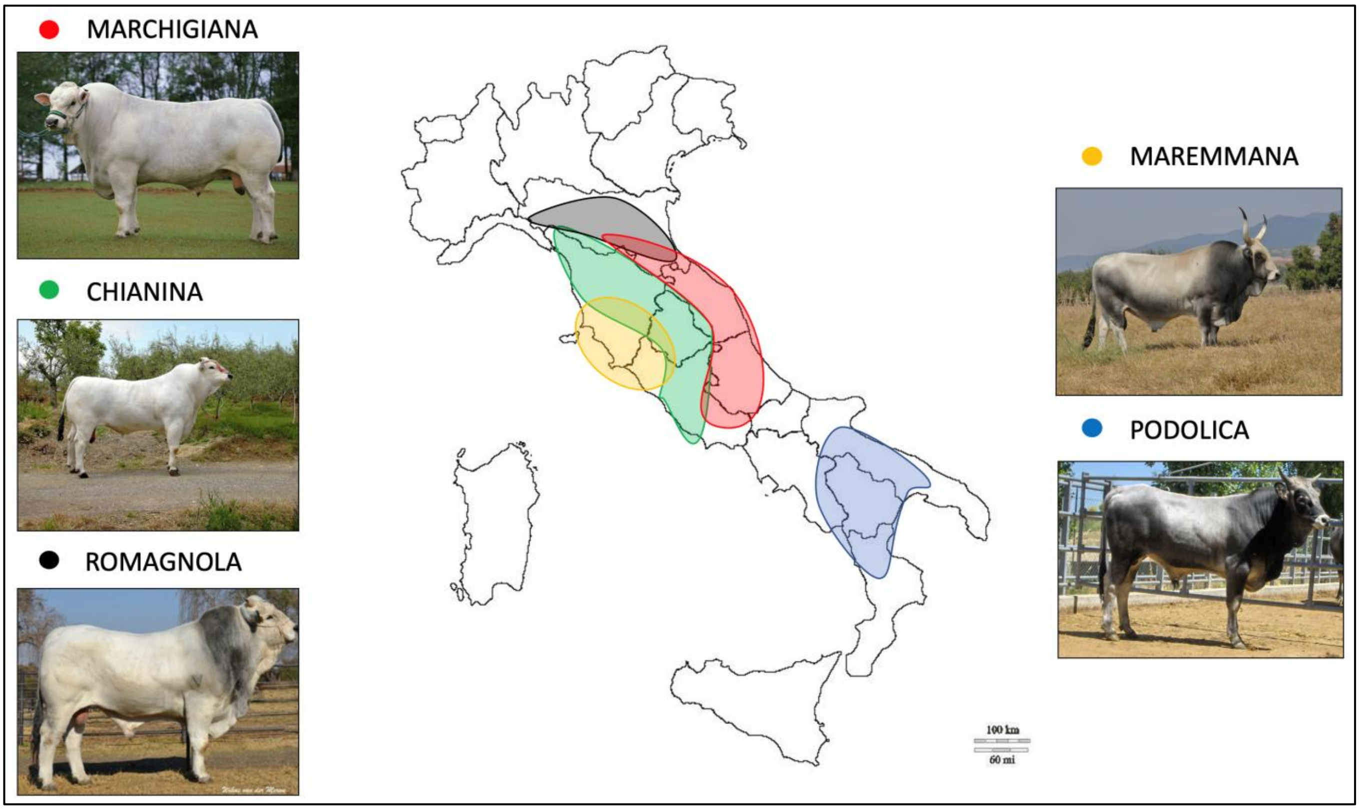

Picture from Rovelli et al., 2021 [1] (revised)

Supplementary Figure S2. Quantile-quantile plot for the GWAS between muscularity and Marchigiana breed (a) and between muscularity and Chianina breed (b).

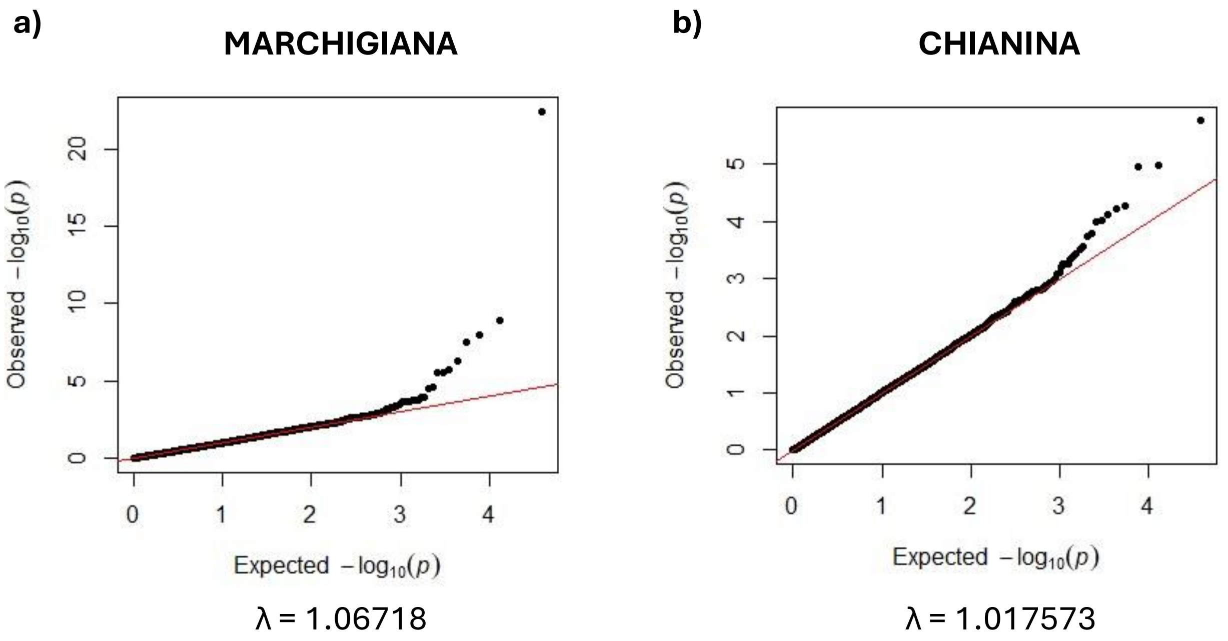

Supplementary Description S1. Morphological and historical description of the five beef cattle breeds (Marchigiana, Chianina, Romagnola, Maremmana, and Podolica) in Italy.

Marchigiana

The Marchigiana breed was improved by crosses with Chianina and Romagnola cattle in the first half of the 19th century [2]. The current Marchigiana breed is the result of a breeding program started after the above-mentioned cross-breeding. Amongst the five breeds investigated in the current work, Marchigiana is the only one showing muscular hypertrophy due to a mutation in the myostatin gene that causes double muscling [3]. Marchigiana has a cylindrical trunk and a light head with short horns. It is characterised by a great precocity, fertility and a remarkable aptitude for beef production. The Herd-book of this breed, at the end of the year 2022, accounted for 50,071 animals [4].

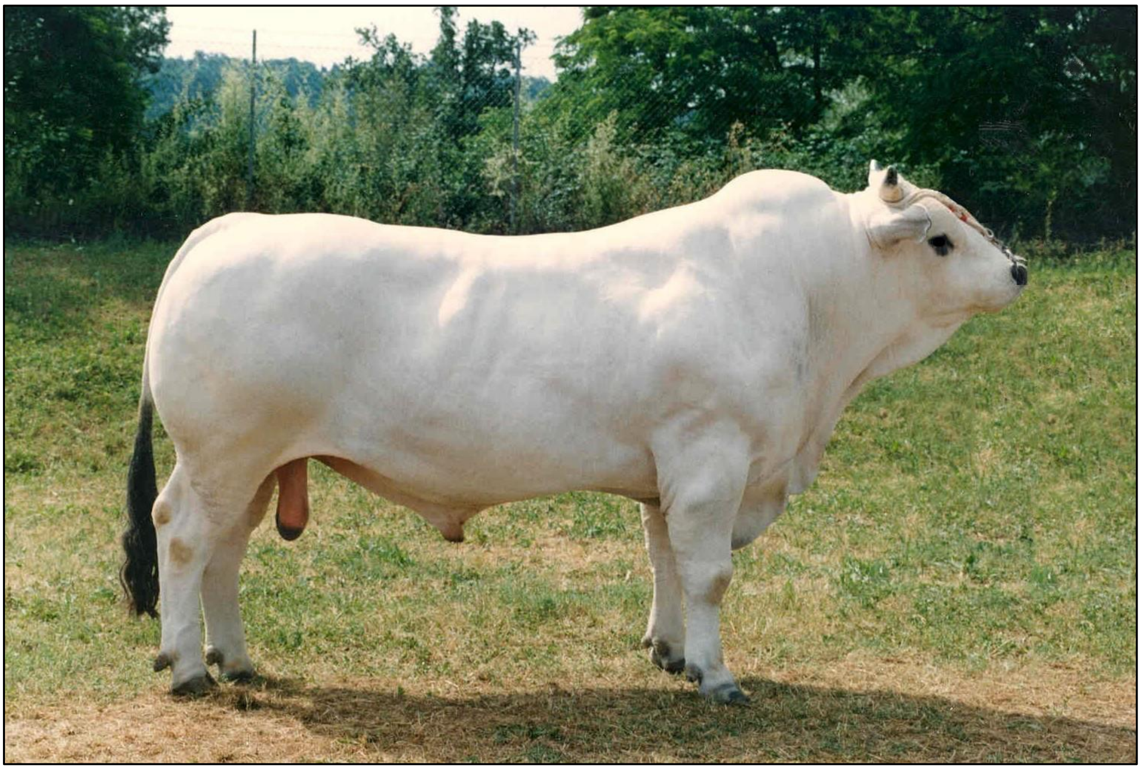

Photo credit: Andrea Quaglia, Fiorella Sbarra - National Association of Italian Beef Cattle Breeders (ANABIC); San Martino in Colle (Perugia, Italy).

Chianina

Chianina is an ancient breed originated in the area of the Val di Chiana (Tuscany region), a fertile valley in central Italy, from which it takes its name [2]. It is a worldwide recognised source of top-quality beef meat, specifically the famous ‘‘Fiorentina steak’’. For these reasons, it has also been exported to countries such as United States of America, Canada, Brazil, Argentina, South Africa, and Australia [5]. Chianina is the tallest of all cattle breeds (measured in height at the withers), and it has a long cylindrical trunk and a solid but light weight skeletal structure. This breed has a fast growth and early maturity, and it is highly adapted to hostile climatic conditions. Chianina was used to improve other breeds, such as Marchigiana [6]. The Herd-book of this breed, at the end of the year 2022, registered 49,274 heads [4].

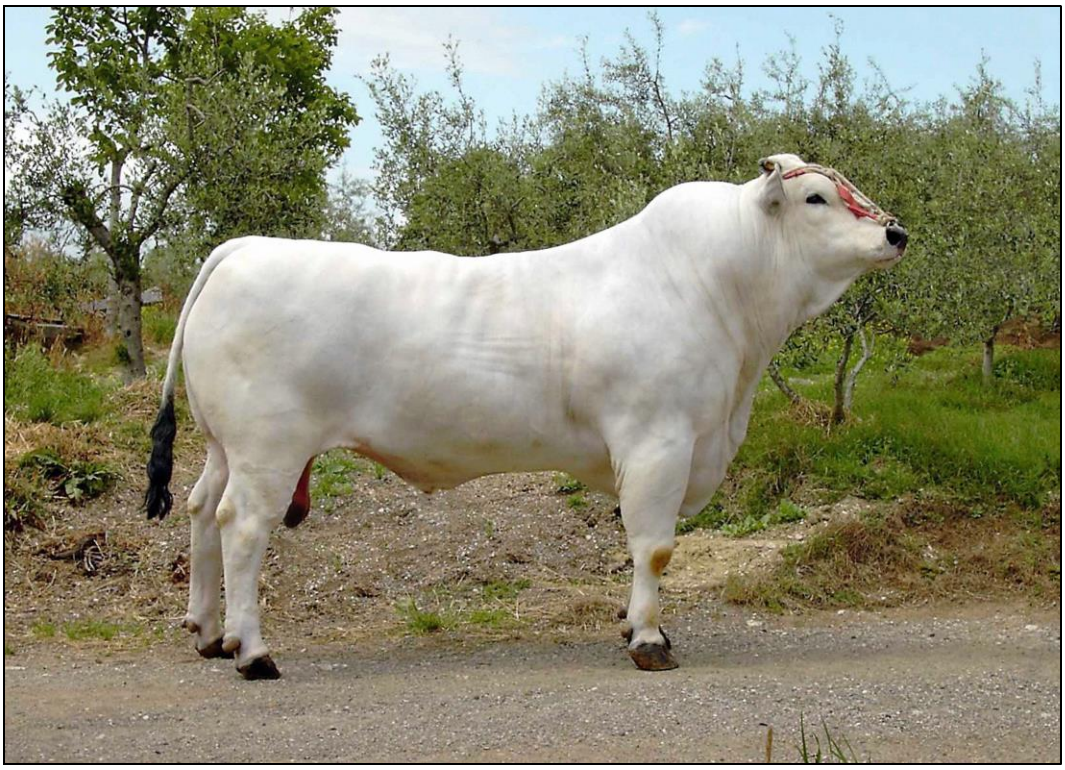

Photo credit: Andrea Quaglia, Fiorella Sbarra - National Association of Italian Beef Cattle Breeders (ANABIC); San Martino in Colle (Perugia, Italy).

Romagnola

Romagnola was originally a dual-purpose breed (work and beef) until the 18th century, but it is only used to produce beef nowadays [2]. Romagnola has been exported to different countries such as United Kingdom, Ireland, Spain, United States of America, Canada, South Africa, and New Zealand [2]. It has an outstanding growth capacity and it shows a brachymorphic structure and well-developed trunk, with strong feet. The Herd-book of this breed, at the end of the year 2022, registered 10,064 heads [4].

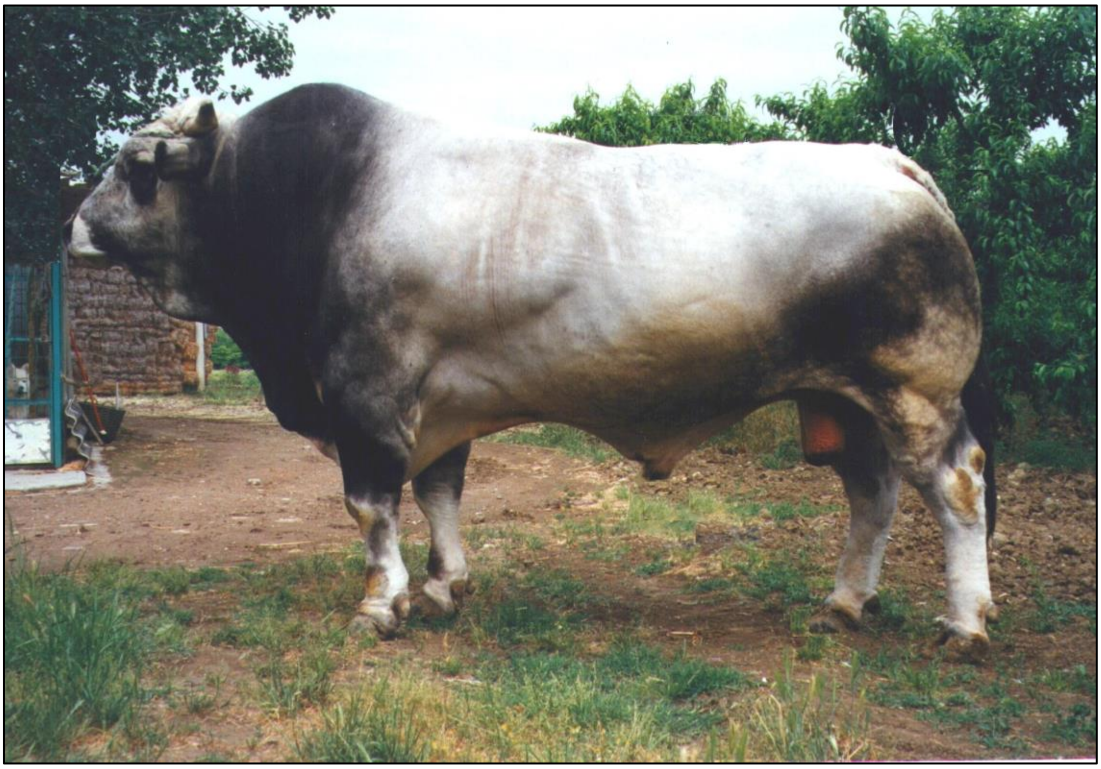

Photo credit: Andrea Quaglia, Fiorella Sbarra - National Association of Italian Beef Cattle Breeders (ANABIC); San Martino in Colle (Perugia, Italy).

Maremmana

Maremmana is a slow-growing Italian beef cattle breed which is selected for adaptability to the marshy land of the Maremma area (central Italy) encompassing Tuscany and Lazio [7]. Maremmana is a rustic breed, characterized by a massive skeletal structure with darker coat and long horns, half-moon-shaped in males and lyre-shaped in females [2, 8]. The Herd-book of this breed, at the end of the year 2022, accounted for 12,262 heads [4].

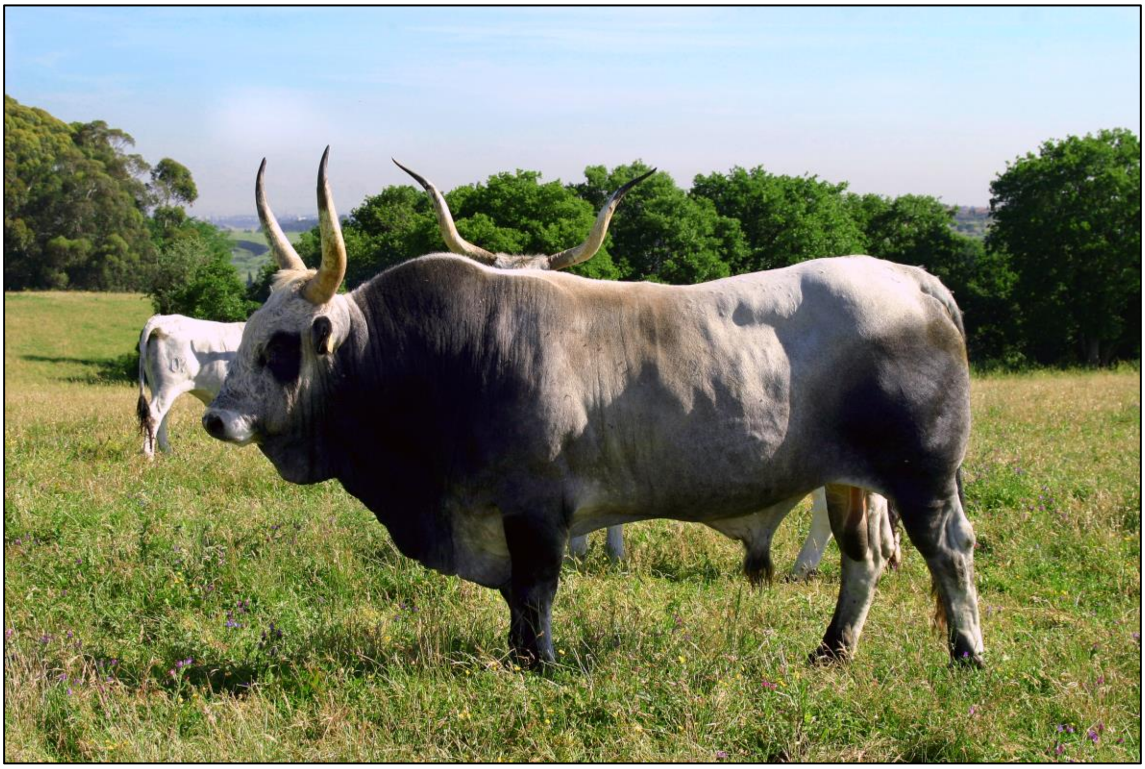

Photo credit: Andrea Quaglia, Fiorella Sbarra - National Association of Italian Beef Cattle Breeders (ANABIC); San Martino in Colle (Perugia, Italy).

Podolica

Podolica is a local breed well adapted to the harsh climatic conditions of southern Italy and which produces high quality milk and meat [7]. It has a lightweight skeletal structure with slender legs and strong feet, and it is well adapted to harsh environments. Even though Podolica is a beef breed, its milk is used to produce the "Caciocavallo" cheese [9]. The Podolica breed accounted for 36,652 heads registered in the Herd-book at the end of the year 2022 [4].

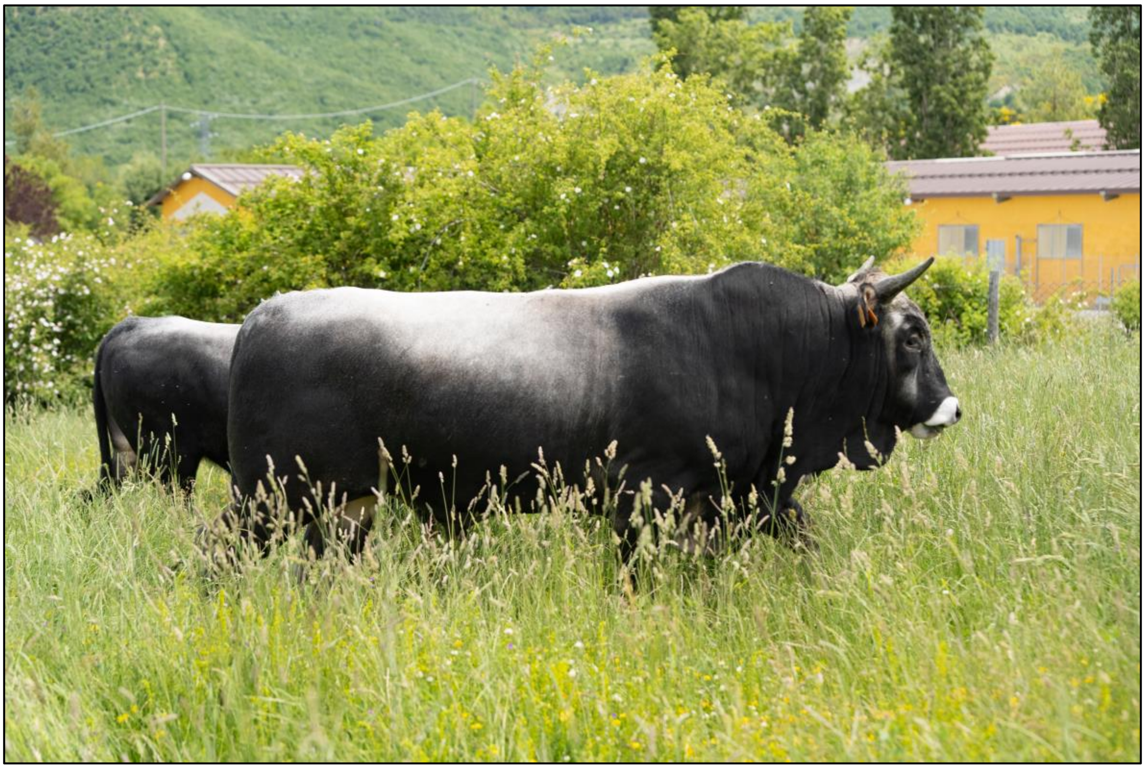

Photo credit: Andrea Quaglia, Fiorella Sbarra - National Association of Italian Beef Cattle Breeders (ANABIC); San Martino in Colle (Perugia, Italy).

References

1. Rovelli, G. *et al.* Evolution of inbreeding: a gaze into five Italian beef cattle breeds history. *PeerJ.* **9**, e12049 (2021).

2. Guarcini, R. Italian beef cattle: current and future situation. *Taurus*. **3,** 39-46 (2009).

3. Sarti, F. M. *et al*. Influence of single nucleotide polymorphisms in the myostatin and myogenic factor 5 muscle growth-related genes on the performance traits of Marchigiana beef cattle. *J. Anim. Sci*. **92,** 3804-3810 (2014).

4. Associazione Nazionale Allevatori Bovini Italiani Carne (ANABIC). http://www.anabic.it/index1.htm (2023). Accessed October 20th, 2023.

5. Sbarra, F. Genetics of autochthonous Italian beef cattle breeds. PhD thesis, 2011. University of Padua (Italy). http://paduaresearch.cab.unipd.it.

6. Guarcini, R. Selezione delle razze bovine da carne: attività e risultati 2012/2015. *Taurus*. **2,** 23-29 (2018).

7. Guarcini, R. Selezione delle razze bovine da carne: attività e risultati 2009/2011. *Taurus*. **6,** 12-57 (2012).

8. Park, S. D. *et al*. Genome sequencing of the extinct Eurasian wild aurochs, Bos primigenius, illuminates the phylogeography and evolution of cattle. *Genome Biol*. **16,** 1-15 (2015).

9. Selvaggi, M., Laudadio, V., D'Alessandro, A. G., Dario, C. & Tufarelli, V. Comparison on accuracy of different nonlinear models in predicting growth of Podolica bulls. *Anim. Sci. J*. **88,** 1128-1133 (2017).
